# Video based hand gesture recognition dataset using thermal camera^☆^

**DOI:** 10.1016/j.dib.2024.110299

**Published:** 2024-03-06

**Authors:** Simen Birkeland, Lin Julie Fjeldvik, Nadia Noori, Sreenivasa Reddy Yeduri, Linga Reddy Cenkeramaddi

**Affiliations:** ACPS Group, Department of Information and Communication Technology, University of Agder, 4879, Norway

**Keywords:** Video hand gestures, Thermal image, Machine learning, Neural network, Jetson nano

## Abstract

The dataset includes thermal videos of various hand gestures captured by the FLIR Lepton Thermal Camera. A large dataset is created to accurately classify hand gestures captured from eleven different individuals. The dataset consists of 9 classes corresponding to various hand gestures from different people collected at different time instances with complex backgrounds. This data includes flat/leftward, flat/rightward, flat/contract, spread/ leftward, spread/rightward, spread/contract, V-shape/leftward, V-shape/rightward, and V-shape/contract. There are 110 videos in the dataset for each gesture and a total of 990 videos corresponding to 9 gestures. Each video has data of three different (15/10/5) frame lengths.

**Specifications Table** This section lists the details of the hardware, the procedure used for collecting the data followed by the format of the data.

**Table:** 

Subject	Human-Computer Interaction, Sign Language Recognition and Translation, Psychology and Neuroscience, Software Engineering, Anthropology and Linguistics, Medical and Rehabilitation Sciences, Robotics and Artificial Intelligence (AI), Education, Music and Performing Arts
Specific subject area	Video of nine different hand gestures represented using hand
Type of data	Video (.avi)
How data were acquired	Thermal camera (FLIR Lepton 2.5, 80×60, 50×, radiometric with shutter) Tripod Stand
Data format	Raw (from acquisition)
Parameters for data collection	Videos for different hand gestures are collected from distinct people with FLIR Lepton Thermal camera placed on a tripod stand in a 3D printed mount
Description of data collection	The camera is connected to a computer via a USB 3.0 port. The camera is connected through a micro USB and an application on our computer that showcases the camera outputs. This application is then used to gather videos. The thermal hand recognition videos were manually stored in their corresponding directories on the computer.
Application scenario	Human-computer interaction, industrial robotics, and automotive user interfaces
Data source location	ACPS group, Department of Information and Communication Technology, University of Agder, Grimstad, Norway, Department of Engineering, University of Cambridge
Data accessibility	Repository Name:
	Thermal Video Dataset of Hand Gestures
	https://zenodo.org/records/10393655

## 1 Value of the Data

• The dataset is useful for developing machine learning to classify and recognize different video-based hand gestures more efficiently.
• The dataset will help computer vision researchers in developing machine learning algorithms for proper classification and recognition of hand gestures.
• The data can be used to create and test new algorithms for video-based hand gesture recognition.
• To incorporate many possible variations in the dataset, data is collected for many different hand gestures at different time instances.

## 2 Background

The majority of datasets available in the literature are captured with an RGB camera. NUS hand digit dataset [1] and ASL Finger Spelling dataset [2] are two of the most commonly used RGB datasets. However, the RGB cameras perform poorly in a variety of lighting conditions [3], [4]. Motivated by this, a thermal imaging dataset has been published in [5] for sign language digits. However, the dataset published in [5] is captured using a low-resolution thermal camera of 32×32 pixels resolution. To address this, a high-resolution dataset has been published in [6]. The dataset in [6] has been collected using a high-resolution thermal camera of 160×120 pixels. The above collected thermal datasets are image-based. Thus, in this work, a video-based hand gesture dataset of 9 classes.

## 3 Data Description

The dataset contains the video frames captured from our thermal camera. The frames are captured from eleven individuals for different hand forms and shapes. The gestures are the same for all 11 individuals. Further, the data is captured by placing the hands at different distances from the thermal camera up to a maximum of 3 meters. The total dataset has been divided into two sections: Classes and takes.

### 3.1 Data file description

**Fig. 1:**
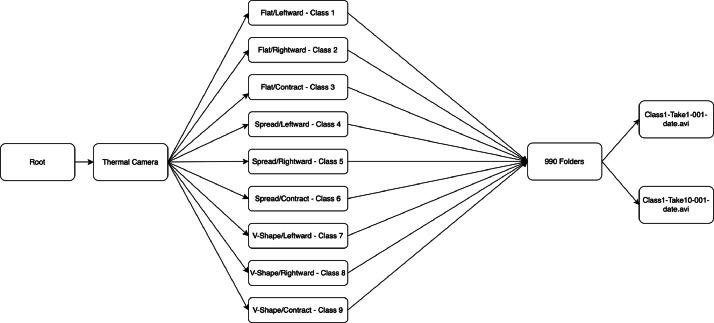
Data structure of the repository.

The layout of the data repository is depicted in Fig. 1. The root folder contains one folder which is divided into 9 folders for each hand gesture. Each hand gesture folder contains video frames captured by 11 different people who did the hand gesture 10 times with different distances and positions in the frame.

**Fig. 2:**
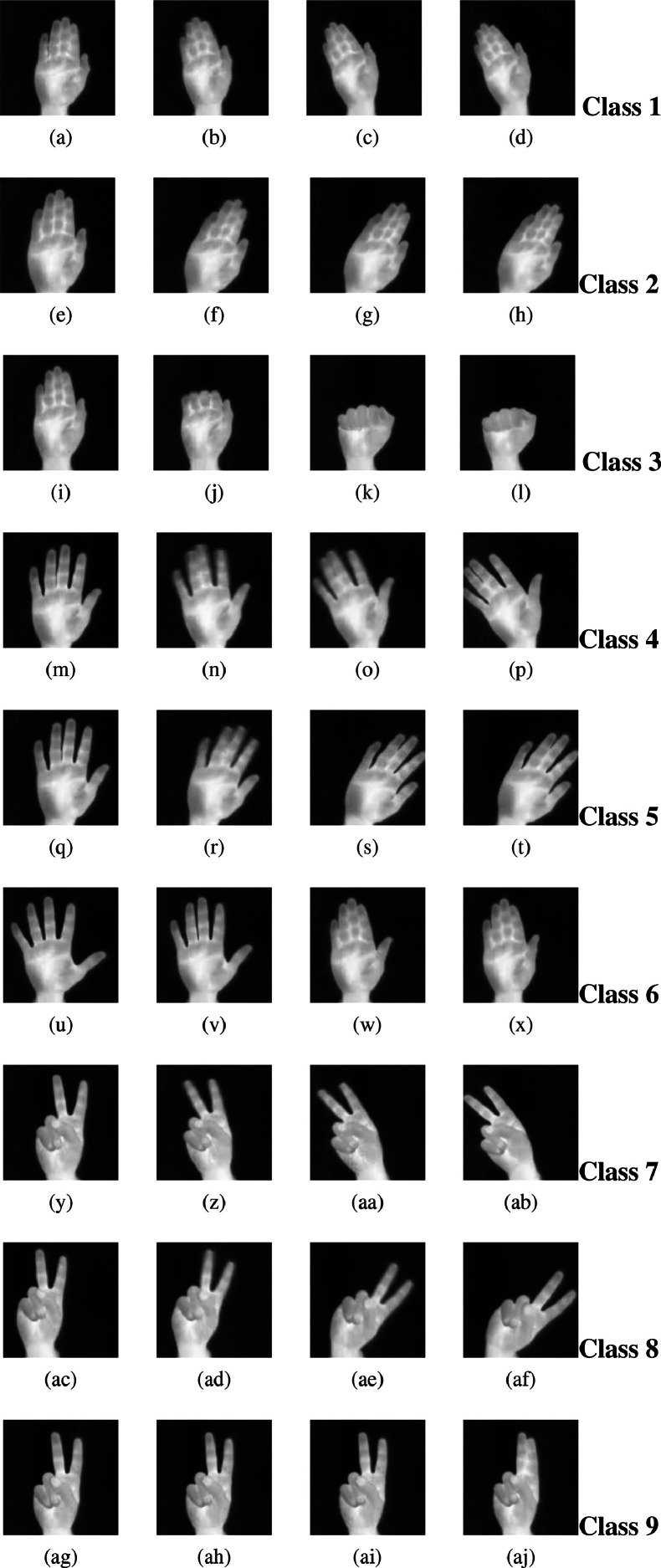
4×9 matrix with all nine classes of hand gestures.

Fig. 2 depicts the complete set of thermal frames captured from the start to the end of the hand position.

Fig. 2 a to d show the frames corresponding to the class 1 Flat/Leftward hand gesture position.

Fig. 2 e to h show the frames corresponding to the class 2 Flat/Rightward hand gesture position.

Fig. 2 i to l show the frames corresponding to the class 3 Flat/Contract hand gesture position.

Fig. 2 m to p show the frames corresponding to the class 4 Spread/Leftward hand gesture position.

Fig. 2 q to t show the frames corresponding to the class 5 Spread/Rightward hand gesture position.

Fig. 2 u to x show the frames corresponding to the class 6 Spread/Contract hand gesture position.

Fig. 2 y to ab show the frames corresponding to the class 7 Vshape/Leftward hand gesture position.

Fig. 2 ac to af show the frames corresponding to the class 8 Vshape/Rightward hand gesture position.

Fig. 2 ag to aj show the frames corresponding to the class 9 Vshape/Contract hand gesture position.

## 4 Experimental Design, Materials and Methods

**Fig. 3:**
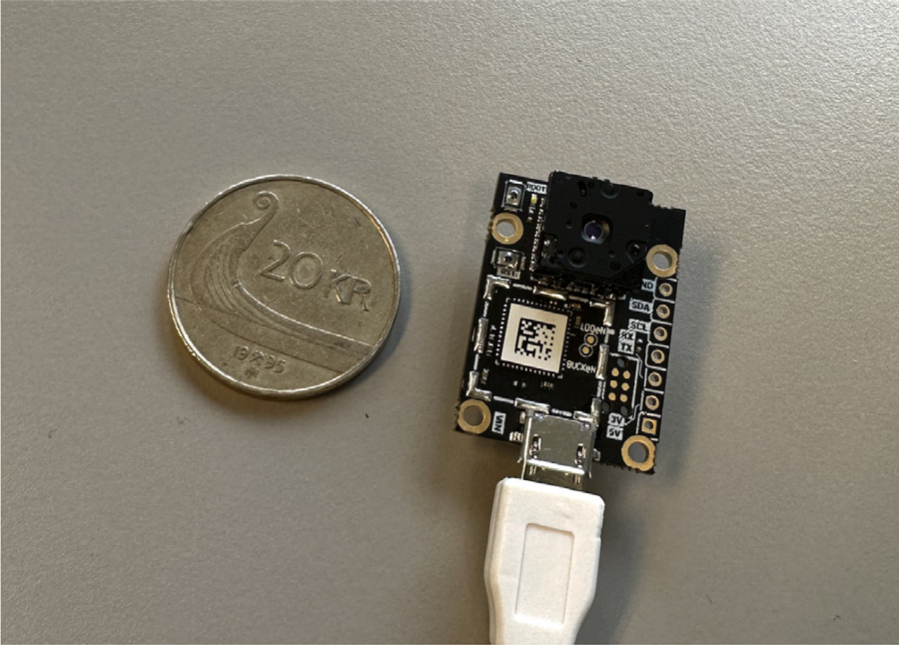
FLIR Thermal Camera next to a 20KR coin.

We used a Thermal camera FLIR Lepton (Lepton 2.5, 80×60, 50×, radiometric with shutter) module as shown in Fig. 3 to capture the hand gestures of an individual. The FLIR Lepton is a radiometric-capable LWIR OEM camera solution that is less than a dime in size, fits inside a smartphone, and costs one-tenth the price of traditional IR cameras. Lepton uses focal plane arrays of either 160×120 or 80×60 active pixels. Every pixel of each image is accurately, calibrated, and noncontact temperature data is captured by the radiometric Lepton [7].

**Fig. 4:**
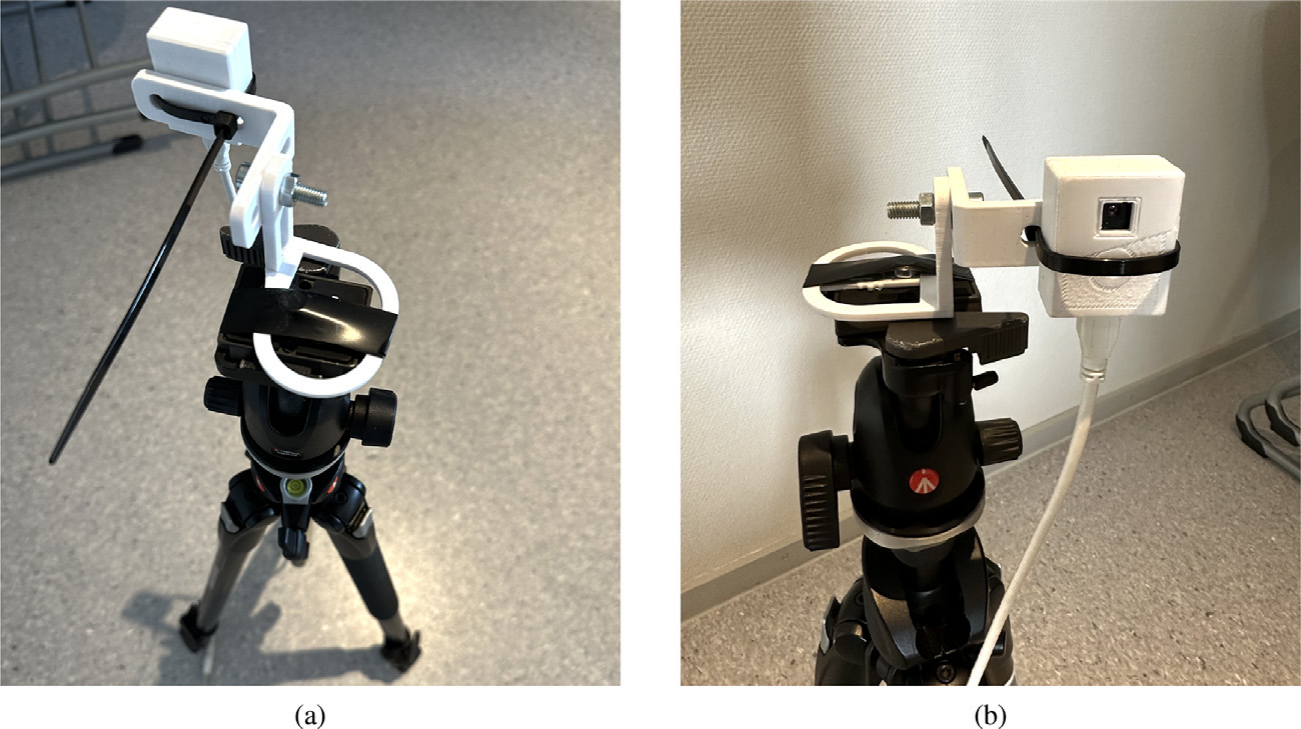
(a) Setup from the back, we can see that the tripod is leveled and (b) Final camera setup.

Fig. 4a shows the setup for capturing the thermal images. Since there is no USB port on the thermal camera, the camera is fitted on purethermal 2 breakout board [8], [9] that has micro USB to connect to the computer through USB. We 3D printed a USB housing for the thermal camera and an adjustable camera stand to mount the camera to the tripod [10], [11] and be able to maintain proper stability. Fig. 4b also shows that we had to use electrical tape and strips to keep the 3D printed parts, the tripod, and the camera together. To collect and save the data, the camera is connected to the HP Laptop 15 computer via USB to micro-USB port to capture the video [12], [13].

## 5 Limitations

While the current model is functioning properly, the use of strips and tape may not provide the best quality or durability for long-term use. Thermal cameras may not be able to capture fine details of hand gestures, which could impact accuracy.

## Ethics Statement

There is no personally identifiable information in the data that was obtained. It is made up of several videos that match the hand gestures. They freely offered their hand gestures as part of the campaign, which was done on people we know and work with.

## CRediT authorship contribution statement

**Simen Birkeland:** Methodology, Software, Data curation, Visualization, Investigation. **Lin Julie Fjeldvik:** Methodology, Software, Data curation, Visualization, Investigation. **Nadia Noori:** Conceptualization, Supervision, Validation, Writing – review & editing, Project administration. **Sreenivasa Reddy Yeduri:** Validation, Writing – review & editing. **Linga Reddy Cenkeramaddi:** Conceptualization, Supervision, Validation, Writing – review & editing, Project administration.

## Data Availability

Thermal Video Dataset of Hand Gestures (Original data) (Zenodo).
